# Prevalence of Carotid Stenosis in Heart Valve Surgery and the Role of Routine Screening

**DOI:** 10.5152/eurasianjmed.2026.251286

**Published:** 2026-04-15

**Authors:** Osman Fehmi Beyazal, Nihan Kayalar, Mehmed Yanartaş

**Affiliations:** Department of Cardiovascular Surgery, İstanbul Başakşehir Çam and Sakura City Hospital, İstanbul, Türkiye

**Keywords:** Carotid stenosis, heart valve, prevalence, screening, stroke

## Abstract

**Background:**

The aim of this study is to determine the prevalence of carotid stenosis (CS) in isolated coronary artery bypass grafting (CABG) and isolated heart valve surgery in the center, identify associated risk factors, and discuss the necessity of routine screening.

**Methods:**

Between 2020 and 2023, a total of 834 patients who had undergone isolated CABG and heart valve surgery were included in the study. The patients were divided into 2 groups: group A (n = 640, isolated CABG) and group B (n = 194, isolated valvular surgery). Subgroups were then created based on age. Group 1 (n = 431, ≥ 60 years), group 2 (n = 371, 40-60 years) and group 3 (n = 32, <40 years). Prevalence of CS, preoperative and postoperative outcomes were compared between groups.

**Results:**

The prevalence of CS ≥ mild, ≥ moderate, and ≥ severe was all higher in group A than in group B. There was no significant difference in cerebrovascular accident (CVA) between groups A and B 9 (1.4%) vs. 1 (0.5%). A positive significant association was found between advanced age (≥ 60 years), CABG, and CS (*OR* = 2.105 and *OR* = 1.899) respectively. Carotid stenosis was not found in any patients under the age of 40. In isolated heart valve surgery patients aged 60 and over, the prevalence of mild or higher CS was 15 (16.9%), moderate or higher CS was 13 (14.6%), and severe or higher CS was 2 (2.2%). In isolated valvular surgery patients aged 40-60, the prevalence of mild or above CS was 5 (6.3%), moderate or above CS was 3 (3.8%), and severe or above CS was 2 (2.5%).

**Conclusion:**

A significant prevalence of CS in patients over the age of 60 undergoing isolated heart valve surgery was found. Screening for CS should be conducted in this patient population, especially in individuals over 60. Screening could also be beneficial for patients over 40, while routine screening is likely unnecessary for those under 40 without cardiovascular risk factors.

**Main Points**

- Routine screening for carotid stenosis (CS) in valvular heart disease is controversial.
- A significant prevalence of CS in patients over the age of 60 undergoing isolated heart valve surgery was found.
- Carotid stenosis screening should be performed especially in individuals over 60 years of age.
- Carotid stenosis screening could also be beneficial for patients over 40 years of age.

## Introduction

Stroke is a significant cause of morbidity and mortality after cardiac surgery. The incidence of postoperative stroke in coronary artery bypass grafting (CABG) patients has been reported to range from 0.5% to 7%.^1^ Mortality in patients who experience a perioperative stroke during CABG increases ninefold.^2^ Carotid stenosis (CS) is one of the most important risk factors for stroke. The incidence of CS in open-heart surgery patients ranges from 3.4% to 22%.^3^ Taneja et al^4^, in their study of neurologically asymptomatic patients undergoing CABG, found a prevalence of CS of 38%. Anselmi et al^5^ reported a prevalence of hemodynamically significant (>50%) CS in valvular heart surgeries of 26.4%. In a meta-analysis by Naylor et al^6^, the risk of stroke was reported to be 7.4% higher in patients with symptomatic/asymptomatic 50%-99% stenosis and 9.1% higher in those with 80%-99% stenosis during cardiac surgery. Therefore, according to the 2024 ESC Guidelines for the Management of Peripheral Arterial Disease,^7^ carotid duplex ultrasound (DUS) is recommended in stable CABG patients who have had a transient ischemic attack (TIA)/stroke within the past 6 months and who have not undergone carotid revascularization (Class 2a). Carotid DUS is recommended with a Class 2b recommendation in stable patients who have not had a TIA/stroke within the past 6 months and are scheduled for CABG.^7^ However, there is currently insufficient evidence for screening in certain populations, such as those with valvular heart disease.

Anselmi et al^5 ^reported that the prevalence of CS in isolated valve surgery is significant, and preoperative screening allows for adjustments to surgical strategy to lower neurological risk. Ascher et al^8^, on the other hand, suggested carotid screening for all patients over 60 years of age undergoing open-heart surgery. While CS screening is not routine in many centers, some centers perform CS screening on all patients undergoing cardiac surgery, regardless of age or risk factors, as seen in Nates et al’s study.^9^ Despite numerous studies on CS screening in CABG and cardiac surgery patients, limited data exist for isolated heart valve surgery, and prevalence rates may vary among populations. Therefore, this study was conducted. The aim of this study is to determine the prevalence of CS in isolated CABG and isolated heart valve surgery in the center, identify associated risk factors, and discuss the necessity of routine screening.

## Material and Methods

This study was designed as a retrospective, single-center observational study that included a total of 834 patients. All patients aged 18 years and older who underwent cardiac surgery at the İstanbul Başakşehir Çam and Sakura City Hospital between 2020 and 2023 were included in the study. Patients who underwent emergency surgeries, aortic dissection, pulmonary thromboendarterectomy, left ventricular assist device implantation, reoperations, perioperative extracorporeal membrane oxygenation, had a history of TIA/cerebrovascular accident (CVA), aortic surgery, those who underwent concomitant carotid endarterectomy (CEA), and those who underwent concomitant CABG/valvular surgery were excluded from the study. The patients were divided into 2 groups: group A (n = 640, isolated CABG) and group B (n = 194, isolated valvular surgery). Subgroups were then created based on age. Group 1 (n = 431, ≥ 60 years), group 2 (n = 371, 40-60 years) and group 3 (n = 32, <40 years).

Medical records of all patients were reviewed including basic demographic characteristics, medical history, preoperative transthoracic echocardiographic (TTE) findings, preoperative laboratory parameters, surgical procedure details, cross clamp (XCL) times, CPB times, intraoperative near infrared spectroscopy (NIRS) values, vasoactive inotrope scores (VIS), the European System for Cardiac Operative Risk Evaluation (EuroSCORE II) score, bleeding amount, CS details, postoperative complications (postoperative exploration, cerebrovascular accident (CVA), postoperative atrial fibrillation (POAF), continuous renal replacement therapy (CRRT) need, intra-aortic balloon pump (IABP), percutaneous coronary intervention (PCI), gastrointestinal bleeding, and mortality. Comparisons were then made between Groups A and B regarding all these parameters. Prevalence comparisons for different CS stenoses were made between groups.

All patients undergoing cardiac surgery at the hospital receive carotid Doppler ultrasound screening. Patients with severe stenosis are further evaluated with carotid computed tomographic angiography (CTA). The classification and treatment recommendations for carotid stenosis, based on the North American Symptomatic Carotid Endarterectomy Trial (NASCET) criteria outlined in the 2024 ESC guidelines, were followed.^7^ Patients with carotid stenosis were categorized as mild (<50%), moderate (50%-69%), severe (≥70%), near occlusion, or total occlusion. Symptomatic carotid stenosis was defined as a transient ischemic attack (TIA) or stroke within the past 6 months related to the ipsilateral carotid stenosis. The presence of carotid intimal thickening and/or plaques without affecting blood flow was considered as the absence of carotid stenosis. Cerebrovascular accident is defined as a neurological deficit that occurred during the entire follow-up period, was confirmed by imaging methods, and validated by a neurologist.

This study was approved by the İstanbul Başakşehir Çam and Sakura City Hospital Ethics Committee (Decision no: 2023-333, July 31, 2023). The study was conducted in accordance with the Declaration of Helsinki. Artificial intelligence-assisted technologies were not used in the production of the submitted work. Patients’ informed consent was obtained for this study.

### Statistics

Statistical analyses were performed using IBM SPSS Statistics for Windows, Version 20.0 (IBM SPSS Corp.; Armonk, NY, USA). Continuous variables in the study were presented as minimum, maximum, median, and interquartile range. Categorical variables were expressed as numbers and percentages. The normality of distribution was assessed by the Kolmogorov–Smirnov test. For numerical variables, differences between patients and controls were tested using *t-*test for parametric data or the Mann–Whitney *U-*test for non-parametric data. Categorical variables were analyzed using the Pearson χ^2^ test and Fisher’s exact test. Multivariate regression analysis was conducted to identify the factors affecting CS. The level of statistical significance was set at *P* < .05.

## Results

The patient’s demographics, comorbidities, TTE findings, and laboratory parameters are presented in Table 1. The mean age was 58.9 ± 10.3 years, with 219 (26.3%) female patients. The mean follow-up period was 422.5 ± 258 days (median: 395, range: 1-1100 days). In group A, female gender was significantly less common than in group B (119 (18.6%) vs. 100 (51.5%), respectively, *P* < .001). The median age was higher in group A than in group B (60 years vs. 58 years, respectively, *P* = .006). Patients’ weight, height, body mass index, and body surface area were higher in group A than in group B. Diabetes mellitus (DM), hypertension (HT), and peripheral artery disease (PAD) were more common in group A. Chronic obstructive pulmonary disease (COPD), preoperative atrial fibrillation, and rheumatic disease were higher in group B. The rates of chronic renal failure (CRF) and operation without discontinuation of dual antiplatelet therapy (DAPT) were similar between the groups. EuroSCORE II was higher in Group B than in Group A (mean 2.7 vs. 1.9, respectively, *P* < .001). Postoperative VIS was also higher in group B than in group A (mean 12 vs. 7.9, respectively, *P* < .001). There was no difference in preoperative ejection fraction and tricuspid annular plane systolic excursion values between the groups. Preoperative white blood cell count, hematocrit, alanine aminotransferase, troponin T, hemoglobin A1c (HbA1c), and low-density lipoprotein (LDL) cholesterol levels were lower in group B than in group A. There were no significant differences between the groups in terms of other laboratory parameters.

The prevalence of isolated mild CS was higher in group A than in group B (43 (6.7%) vs. 4 (2.1%), respectively, *P* = .01). The rates of isolated moderate, severe, near-occlusion, and total occlusion were similar between the groups. Additionally, the prevalence of CS ≥mild, ≥moderate, and ≥severe was all higher in group A than in group B (150 (23.4%) vs. 20 (10.3%), *P* = .001, 107 (16.7%) vs. 16 (8.2%), *P* = .004, and 46 (7.2%) vs. 4 (2.1%), *P* = .008, respectively).

Operative data, amount of bleeding, and NIRS values are presented in Table 2. cross clamp and CPB times were longer in group B than in group A (mean, 76 min vs. 101 min, *P* < .001, and 122 min vs. 144 min, *P* = .001, respectively). The mean number of bypass grafts in group A was 3. In group A, the left internal thoracic artery was used in 573 (89.5%) patients, beating surgery was performed in 56 (8.8%), and coronary endarterectomy was performed in 27 (4.2%). In group B, 20 (10.3%) patients underwent ablation surgery, and 12 (6.2%) patients were operated on for infective endocarditis. No significant difference was found between the groups in terms of NIRS values before XCL and after XCL at 30 minutes. The amount of postoperative bleeding was higher in group A than in group B (mean 812 mL vs. 638 mL, respectively, *P* < .001).

Postoperative outcomes are presented in Table 3. There was no significant difference in CVA between groups A and B (9 (1.4%) vs. 1 (0.5%), respectively, *P* = .28). Postoperative exploration and CRRT requirement were higher in group B than in group A (23 (11.9%) vs. 35 (5.5%), *P* = .002 and 12 (6.2%) vs. 14 (2.2%), *P* = .005, respectively). No significant differences were found between the groups in terms of POAF, IABP, gastrointestinal bleeding, and PCI requirement. Similarly, total mortality rates were similar between the groups (Group A: 19 (3%) vs. group B: 8 (4.1%), *P* = .42).

The results of the multivariate regression performed to determine the factors affecting CS are presented in Table 4. A positive significant association was found between advanced age (≥60 years), CABG, and CS (*P* < .001, *OR* = 2.105 (95% CI, 1.390-3.186) and *P* = .03, *OR* = 1.899 (95% CI, 1.046-3.449), respectively). No significant association was found between female gender, DM, HT, COPD, CRF, and CS.

**Figure 1.**
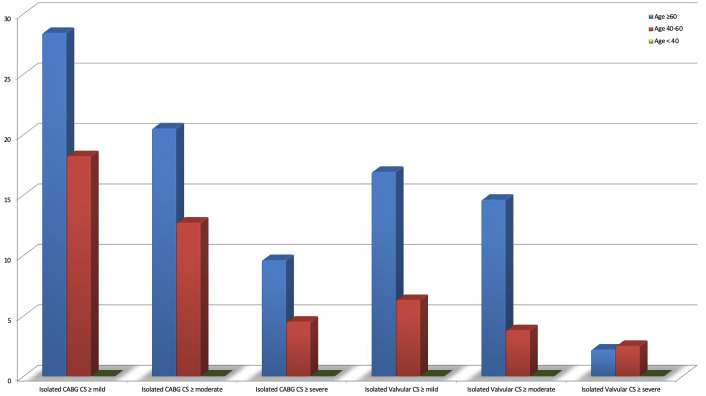
Comparison of carotid stenosis prevalence among subgroups divided by age.

The results of subgroup analysis performed by age group are presented in Table 5. In the study group, CS was not found in any patients under the age of 40. For isolated CABG patients aged 60 and over, the prevalence of mild or higher CS was 97 (28.4%), moderate or higher CS was 70 (20.5%), and severe or higher CS was 33 (9.6%). In isolated valvular surgery patients aged 60 and over, the prevalence of mild or higher CS was 15 (16.9%), moderate or higher CS was 13 (14.6%), and severe or higher CS was 2 (2.2%). It was found that in isolated CABG patients aged 40-60, the prevalence of mild or above CS was 53 (18.2%), moderate or above CS was 37 (12.7%), and severe or above CS was 13 (4.5%). In isolated valvular surgery patients aged 40-60, the prevalence of mild or above CS was 5 (6.3%), moderate or above CS was 3 (3.8%), and severe or above CS was 2 (2.5%) (Figure 1).

## Discussion

Stroke is a significant cause of morbidity and mortality following cardiac surgery, with risk factors including advanced age, DM, HT, smoking, gender, PAD, atherosclerosis, CPB, and cardiac surgery itself.^10^ The incidence of stroke is higher in cardiac surgery compared to the general population. Carotid stenosis has been reported in approximately 20% of patients with severe coronary artery disease (CAD), approximately 22% in the presence of HT and cardiac disease, 14% in those over the age of 60 with 2 cardiovascular risk factors (CVRFs) (HT, CAD, current smoking, first-degree family history of stroke), 16% in those with 3 CVRFs, and approximately 67% in those with 4 CVRFs.^11^ ^-^^13^ Coexistence is more common, especially in patients with CAD, due to similar pathophysiological processes. In contrast, coexistence is not as common in valvular heart disease as it is in CABG due to the different pathophysiological mechanisms (rheumatic, degenerative, functional, etc.). However, because this patient group shares similar cardiac risk factors, recognizing the presence of CS and implementing strategies to address it can reduce postoperative stroke rates. For this reason, some clinics, like the present one, perform screening for CS in cardiac surgery patients.^2^^,^^9^ However, there is currently insufficient evidence to support the routine use of this screening for all patients or a specific age group. Therefore, studies are needed to determine the prevalence of CS in this patient group and compare it across different age groups. This is why this study was designed. The findings indicate a significant prevalence of CS in isolated heart valve surgery patients, particularly those over the age of 60.

To investigate the prevalence of CS in heart valve surgery, rigorous selection criteria was applied to obtain a group with isolated valvular surgery. To avoid the influence of atherosclerosis pathogenesis, patients who underwent concomitant CABG were excluded. Also, concomitant CEA procedures and other cardiac procedures were excluded due to the risk of confounding the CVA results. Therefore, the prevalence of CS in a group of patients undergoing isolated valvular surgery without a history of TIA/stroke were investigated. Also, this prevalence was compared with the isolated CABG group. As expected, these rates were found to be higher in isolated CABG patients at 23.4% and 16.7%, respectively. However, these rates may vary among populations. In a Bangladeshi ischemic heart disease cohort by Ranjan et al, the prevalence of CS was 13.5%; in CABG patients by Taneja et al. in India, the prevalence of CS was 38%; and in a study by Chen et al, the prevalence of CS with stenosis of 50% or more was 19.2%, and the prevalence of CS with stenosis of 70% or more was 6.9%.^4^^,^^14^^,^^15^ In the study, it was found that the prevalence of CS in isolated CABG patients is similar to that in other studies. The relatively high rates of CS in comparison to the general population have led to the inclusion of carotid DUS screening in guidelines for this patient group.^7^ However, while numerous studies have examined the prevalence of CS in cardiac surgery patients, there is little research in valvular surgery patients. An Italian study by Anselmi et al^5^ reported a CS with >50% stenosis in valvular heart surgery patients at 26.4%, while Lepidi et al’s^16^ study on transcatheter aortic valve implantation (TAVI) patients found a CS prevalence with >70% stenosis at 9%. In the study, the prevalence of moderate CS (over 50%) was 8.2%, and severe CS (over 70%) was 2.1%. Given that TAVI patients typically have more comorbid conditions, it can be concluded that the prevalence of CS in this study is not insignificant.

Another important point to consider is the distinction between age groups. The multivariate analysis revealed that individuals over the age of 60 had an independent risk factor for CS (OR: 2.1). When the patients were divided into subgroups, it was found that the prevalence of CS above moderate was 14.6% in isolated valvular surgery patients over the age of 60, while the prevalence of CS above severe was 2.2%. Additionally, any cases of CS in patients under the age of 40 was not found, including those in the CABG group. These findings suggest that the prevalence of CS is higher in the older age group and that screening for CS should be conducted in this population. However, it can be concluded that routine screening is unnecessary for patients under the age of 40 without CVRFs.

In the study group, it was found that patients in Group A had a higher prevalence of comorbid conditions closely associated with atherosclerosis, such as DM, HT, and PAD. Troponin T, LDL cholesterol, and HbA1c levels were also higher in group A, indicating a correlation with these pathologies. Additionally, it was observed that rheumatic diseases and preoperative atrial fibrillation were more common in group B, which aligns with the pathophysiological mechanisms of valvular disease. EuroSCORE II was also higher in group B. Group A consisted of patients undergoing isolated CABG procedures, while group B included patients undergoing multiple valve procedures. Due to the higher number of procedures in group B, which included 3 valve procedures and ablation surgery, longer XCL and CPB times were found. Furthermore, postoperative drainage was significantly higher in the CABG group, despite a shorter CPB duration. One of the reasons for this discrepancy could be attributed to the fact that CABG patients in the clinic are routinely operated on while receiving acetylsalicylic acid. However, in group B, unless they have preoperative atrial fibrillation, they are not receiving any antiplatelet/anticoagulant therapy. Additionally, although not statistically significant, a higher number of patients in the CABG group were operated on with dual antiplatelet therapy (DAPT), which could also contribute to increased drainage.

Regarding postoperative outcomes, the need for postoperative exploration and CRRT was higher in group B. Procedures such as longer XCL and CPB times, triple-valve procedures, and concomitant ablation surgery may have played a role. However, there were no significant differences between the groups in terms of mortality and other postoperative outcomes. There were no significant differences in intraoperative pre-XCL and XCL 30-minute NIRS values between the groups. Similarly, no difference in CVA was found. Cerebrovascular accident was observed in 1.4% of isolated CABG patients. Gaudino et al’s^17^ study reported a 1.3% incidence of stroke after CABG. The CVA rate in the isolated CABG group in the study is similar to the literature. Alwaqfi et al^18^ reported that stroke after heart valve surgery ranges from 1 to 10%. In the study, only 1 patient (0.5%) in the isolated valve surgery group had CVA. This lower rate, compared to the literature, may be related to close screening for CS, although not solely. Although >50% stenosis is considered significant for CS, 16.9% rate of pathology in this study was found, especially when <50% stenosis was included in patients over 60 years of age. These lesser-grade plaques in the extracranial carotid arteries can progress to critical CS over time. Furthermore, these stenoses, which are not considered hemodynamically critical, can pose a risk for stroke during hypothermic CPB when hemodynamic and physiological characteristics change. The extent to which these noncritical stenoses pose a risk for CVA remains uncertain. However, it is thought that the prevalence of CS in patients over 60 years of age, as found in the study, is significant. Identifying critical or noncritical carotid artery stenoses can alter operative strategies and lifestyle changes, plan optimal medical treatment with lipid-lowering agents such as statins, and reduce postoperative stroke rates. Therefore, based on the findings in this study, it is thought that carotid screening should be performed in all patients over 60 years of age during valvular heart surgery. Screening may be beneficial in patients over 40 years of age, but routine screening is not necessary in patients under 40 years of age without CVRFs.

### Limitations

The first limitation of this study is its retrospective and single-center nature. Secondly, because patients did not undergo routine CT scans, factors that could pose a risk for CVA, such as aortic plaques and calcifications, were not compared between the groups. Screening for CVA was conducted with carotid DUS, while CTA was only performed for critical lesions. It is important to note that DUS can yield false-positive/negative results and may not accurately depict true stenosis in all patients. Although DUS is routinely performed in the clinic, a cost-effectiveness analysis was not conducted in this study. However, it would be beneficial to include this analysis in future studies.

## Conclusion

In this retrospective study, a significant prevalence of CS was found in patients over the age of 60 undergoing isolated heart valve surgery. Screening for CS should be conducted in this patient population, especially in individuals over 60. Screening could also be beneficial for patients over 40, while routine screening is likely unnecessary for those under 40 without CVRFs. Nevertheless, further prospective studies are needed to confirm these findings.

## Figures and Tables

**Table 1.** Comparison of Patient Demographics, Comorbidities, Echocardiographic Findings, and Laboratory Parameters Between Group A and Group B

	**Group A (n = 640)** **Isolated CABG**			**Group B (n = 194)** **Isolated Valvular**			*P*
	**Min-Max or n (%)**	**Median (Mean)**	**IQR**	**Min-Max or n (%)**	**Median (Mean)**	**IQR**	
Demographic data							
Gender female	119 (18.6)			100 (51.5)			**<.001**
Age (years)	25-86	60	13	19-78	58	20	**.006**
Height (cm)	137-196	169.5	10	140-203	165	13	**<.001**
Weight (kg)	53-130	80	17	39-130	76	18	**.002**
Body surface area (kg/m^2^)	1.44-2.5	1.9	0.21	1.22-2.58	1.83	0.25	**<.001**
Body mass index (m^2^)	16.9-55.6	27.8	5.5	17.7-49	27.7	6.7	.28
Comorbid diseases							
CS mild <50%	43 (6.7)			4 (2.1)			**.01**
CS moderate 50%-69%	61 (9.5)			12 (6.2)			.14
CS severe ≥70%	40 (6.3)			4 (2.1)			.02
CS near-occlusion	1 (0.2)			0			.76
CS total occlusion	5 (0.8)			0			.26
CS ≥ mild	150 (23.4)			20 (10.3)			**.001**
CS ≥ moderate	107 (16.7)			16 (8.2)			**.004**
CS ≥ severe	46 (7.2)			4 (2.1)			**.008**
Diabetes mellitus	320 (50.6)			47 (24.2)			**<.001**
Hypertension	397 (62)			13 (6.7)			**<.001**
Chronic obstructive pulmonary disease	43 (6.7)			23 (11.9)			**.02**
Preoperative atrial fibrillation	7 (1.1)			43 (22.2)			**<.001**
Chronic renal failure	32 (5)			13 (6.7)			.22
Peripheral artery disease	39 (6.1)			3 (1.5)			**.01**
Rheumatic disease	10 (1.6)			14 (7.2)			**<.001**
Operation with dual antiplatelet	19 (3)			2 (1)			.13
EuroSCORE II	1-18	1 (1.9)	1	1-28	2 (2.7)	2	**<.001**
Vasoactive inotropic score (VIS)	0-300	0 (7.9)	5	0-240	3.5 (12)	10	**<.001**
Echocardiographic findings							
Preop ejection fraction (%)	20-65	55	10	20-65	60	10	.30
Preop TAPSE (mm)	11-33	23	4	13-41	22	6	.29
Preop laboratory parameters							
White blood cells (10^9^/L)	3.2-22	8.5	11	1.3-23.7	7.7	2.9	**<.001**
Hematocrit (%)	20.5-58.7	41	6	22.5-53	39	7	**<.001**
Platelets (10^9^/L)	74-586	253	89	92-772	241	94	.05
Urea (mg/dL)	7.8-272	33.7	16	9.3-113.7	34.6	17.3	.57
Creatinine (mg/dL)	0.27-11.8	0.93	0.32	0.49-5.38	0.89	0.27	.13
Sodium (mEq/L)	124-150	139	4	120-149	140	4	.10
Potassium (mEq/L)	2.69-6.37	4.38	0.6	3.25-5.98	4.47	0.52	.10
Alanine aminotransferase (IU/L)	1-222	19	12	4-202	17	13	**.01**
Aspartate aminotransferase (IU/L)	5-431	20	11	8-118	19	9	.08
C-reactive protein (mg/dL)	0.2-211.2	3.6	6.8	0.1-277	3.6	8.4	.85
Troponin T (ng/mL)	1-1860	14.7	31.6	2-1994	12.4	12.2	**<.001**
HbA1c (mmol/mol)	2.9-16.2	6.3	2.1	4.2-8.7	5.8	0.7	**<.001**
Ldl cholesterol (mg/dL)	24-377	107	58	30-208	95	50	**.005**

CABG, coronary artery bypass graft; CS, carotid stenosis; EuroSCORE II, The European System for Cardiac Operative Risk Evaluation; IQR, interquartile range; TAPSE, tricuspid annular plane systolic excursion.

Bold values indicate statistical significance

**Table 2.** Comparison of Operative Data and Bleeding Amounts, Between Group A and Group B

	**Group A (n = 640)** **Isolated CABG**			**Group B (n = 194)** **Isolated Valvular**			
	**Min-Max or n (%)**	**Median (Mean)**	**IQR**	**Min-Max or n (%)**	**Median (Mean)**	**IQR**	***P***
Operative data							
Cross-clamp time (min)	0-217	76 (76)	49	0-317	98.5 (101)	59	**<.001**
Cardiopulmonary bypass time (min)	0-407	123 (122)	59	0-391	138 (144)	65	**.001**
The number of grafts	1-7	3	2	–			–
LITA usage	573 (89.5)			–			–
Beating heart	56 (8.8)			–			–
Coronary endarterectomy	27 (4.2)			–			–
Infective endocarditis	–			12 (6.2)			–
Ablation	–			20 (10.3)			–
Before XCL NIRS right (%)	33-99	62	11	38-85	61	12	.11
Before XCL NIRS left (%)	30-94	62	9	31-86	61	13	.09
After XCL NIRS right (%)	33-94	59	8	37-87	59	11	.37
After XCL NIRS left (%)	23-83	59	10	31-95	59	11	.46
Postop total amount of bleeding (mL)	100-3250	700 (812)	400	50-3600	550 (638)	400	**<.001**

IQR, interquartile range; LITA, left internal thoracic artery; NIRS, near-infrared spectroscopy; XCL, cross-clamp.

Bold values indicate statistical significance

**Table 3.** Comparison of Postoperative Data Between Group A and Group B

	**Group A (n = 640)** **Isolated CABG** **n (%)**	**Group B (n = 194)** **Isolated Valvular** **n (%)**	*P*
Cerebrovascular accident	9 (1.4)	1 (0.5)	.28
Postoperative exploration	35 (5.5)	23 (11.9)	**.002**
Continuous renal replacement therapy	14 (2.2)	12 (6.2)	**.005**
Postop atrial fibrillation	97 (15.2)	35 (18)	.33
Intra aortic balloon pump	7 (1.1)	0	.15
Percutaneous coronary intervention	6 (0.9)	0	.20
Gastrointestinal bleeding	3 (0.5)	4 (2.1)	.055
Mortality	19 (3)	8 (4.1)	.42

CABG, coronary artery bypass grafting.

Bold values indicate statistical significance

**Table 4.** Multivariate Logistic Regression Analysis

	**Odds Ratio**	**CI 95%**	*P*
Gender female	0.752	0.454-1.247	.26
Age	**2.105**	1.390-3.186	**<.001**
Diabetes Mellitus	1.272	1.046-3.449	.26
Hypertension	0.980	0.631-1.523	.92
COPD	1.289	0.657-2.529	.46
Chronic renal failure	1.154	0.513-2.597	.72
CABG	**1.899**	1.046-3.449	**.03**

CABG, coronary artery bypass graft; COPD, chronic obstructive pulmonary disease.

Bold values indicate statistical significance.

**Table 5.** Comparison of the Prevalence of Carotid Stenosis in Subgroups Separated by Age

	**Group 1 (n = 431)** **Age ≥60** **n (%)**	**Group 2 (n = 371)** **Age 40-60 ** **n (%)**	**Group 3 (n = 32)** **Age < 40** **n (%)**	***P***
Isolated CABG	(n = 342)	(n = 291)	(n = 7)	
CS ≥ mild	97 (28.4)	53 (18.2)	0	**.04**
CS ≥ moderate	70 (20.5)	37 (12.7)	0	**.01**
CS ≥ severe	33 (9.6)	13 (4.5)	0	**.03**
Isolated valvular	(n = 89)	(n = 80)	(n = 25)	
CS ≥ mild	15 (16.9)	5 (6.3)	0	**.01**
CS ≥ moderate	13 (14.6)	3 (3.8)	0	**.01**
CS ≥ severe	2 (2.2)	2 (2.5)	0	.73

CABG, coronary artery bypass graft; CS, carotid stenosis; IQR: interquartile range.

Bold values indicate statistical significance.
